# Combining Instruction Prefetching with Partial Cache Locking to Improve WCET in Real-Time Systems

**DOI:** 10.1371/journal.pone.0082975

**Published:** 2013-12-26

**Authors:** Fan Ni, Xiang Long, Han Wan, Xiaopeng Gao

**Affiliations:** School of Computer Science and Engineering, Beihang University, Beijing, China; National Microelectronics Center, Spain

## Abstract

Caches play an important role in embedded systems to bridge the performance gap between *fast* processor and *slow* memory. And prefetching mechanisms are proposed to further improve the cache performance. While in real-time systems, the application of caches complicates the Worst-Case Execution Time (WCET) analysis due to its unpredictable behavior. Modern embedded processors often equip locking mechanism to improve timing predictability of the instruction cache. However, locking the whole cache may degrade the cache performance and increase the WCET of the real-time application. In this paper, we proposed an instruction-prefetching combined partial cache locking mechanism, which combines an instruction prefetching mechanism (termed as BBIP) with partial cache locking to improve the WCET estimates of real-time applications. BBIP is an instruction prefetching mechanism we have already proposed to improve the worst-case cache performance and in turn the worst-case execution time. The estimations on typical real-time applications show that the partial cache locking mechanism shows remarkable WCET improvement over static analysis and full cache locking.

## Introduction

Cache memories are often used in embedded systems to bridge the performance gap between *fast* processors and *slow* memory systems by exploiting the temporal and spatial locality of memory accesses in a program. However, in hard real-time systems, the cache is problematic due to its unpredictable behavior when calculating the Worst-Case Execution Time (WCET) of a program. WCET is a very important metric when performing the schedulability analysis of a real-time system. It is defined as the upper bound on the execution time on a given hardware platform across all possible inputs. The execution time of each basic block through the worst-case execution path has to be calculated to get the WCET of a program, which in turn depends on determining the time spent on each instruction in the basic blocks. In the presence of caches, the execution time of an instruction may vary due to hit or miss of the access to the instruction cache, which brings in significant imprecision in WCET analysis.

To improve time predictability of caches, modern embedded processors often feature a cache locking mechanism. In the cache locking mechanism, selected memory blocks are loaded into the cache in advance (in static cache locking [1]) or at given program points (in dynamic cache locking [2] [3]). Once a memory block is locked, it will be kept in the cache without being evicted by the replacement policy. An access to the memory blocks locked is determined as a hit, otherwise a miss is determined. In this way, the timing predictability of the cache accesses is achieved. However, locking the whole cache may dramatically increase the WCETs of the real-time programs. In [4], the author gives an example to explain it. Fortunately, embedded systems often provide options of partial cache locking through way locking and line locking. Particular ways are locked entirely for all cache sets in way locking, while line locking allows locking a different number of blocks in different sets. In partial cache locking, some blocks are locked and the remaining unlocked portion acts just as a normal cache.

In previous studies, we proposed an instruction prefetching mechanism performed at the basic block level (termed as BBIP) to improve the worst-case cache performance. A paper related appears on PDCAT 2012 [5]. In this paper, we are trying to combine the BBIP mechanism with partial cache locking to enforce their performance on improving WCET estimation.

The contributions of the paper can be concluded as follows:

- A partial cache locking mechanism is integrated with BBIP to improve both the worst-case cache performance and the WCETs of real-time applications.
- A greedy two-phase content selection algorithm is proposed to select the most *significant* basic blocks to lock in the cache to provide sub-optimal performance.
- Detailed evaluation and analysis of the improvement of the BBIP-combined partial cache locking mechanism over static analysis and full cache locking on typical real-time benchmarks are presented.

## Related Work

Cache locking is a common approach to improve the predictability of the instruction cache in real-time systems. It loads some contents in advance and locks them in order to ensure they are never swapped out by the replacement policy until they are unlocked. The ability to lock contents is available in some commercial processors, such as Motorola ColdFire MCF5249, Motorola PowerPC 440, Motorola MPC7451, MIPS32, ARM 940 and ARM 946E-S. The performance of cache locking on predictability lies heavily on the contents locked in the cache, which are selected by the content selection algorithm. Currently, most cache locking technologies are proposed to select cache contents to fill in the entire cache (termed *full cache locking*), which means no replacement is allowed to be performed on the cache during the access. Typical cache content selection algorithms for full cache locking are proposed in [6] [7] [8] [9] [10] [11]. Full cache locking works well to improve the predictability of the cache behaviour, which is of great importance when the static analysis technology is not so effective. As state-of-the-art instruction cache modelling technologies are very mature and most instruction access can be classified statically as hit/miss, recent research [4] reveals that it is not always a good idea to lock the entire cache in order to minimize the WCET and in some cases, leaving part of the cache unlocked and acting as a normal cache leads to a lower WCET estimate. To the best of our knowledge, [4] is the only work published on partial cache locking to improve the WCETs of the real-time applications.

Instruction prefetching technologies are proposed to improve the cache performance, especially the average-case cache performance. And very few studies have focused on the impact of the instruction prefetching on WCET analysis and the performance of real-time applications. In [12], Lee et al. presented the WCET analysis for a buffered prefetching scheme based on timing schema, which is more like a new architecture to replace the instruction cache, rather than a new feature attached to it. Jun Yan and Wei Zhang [13] investigated the impact of the Next-N-Line instruction prefetching on the worst-case execution time. And their following work [14] proposed a loop-directed prefetching and a WCET-oriented software prefetching. Our previous work [5] proposed an instruction prefetching technology performed at the basic block level (termed BBIP), which is designed to improve both the worst-case cache performance and WCETs of real-time applications. The experiments showed that BBIP not only works well in reducing the worst-case cache misses, but also improves the WCET.

Luis C. Aparicio et al. [15] combined instruction prefetching with instruction cache locking in multitasking real-time systems to improve the WCET estimations. It is the only work we can find published in the area. Our work presented in this paper is different from it in several ways. Firstly, the prefetching mechanism we used here is more aggressive, which results in a design of the content selection algorithm from scratch. In [15], the *next-Line tagged* prefetching performed at the memory block level is considered, while BBIP performed at the basic block level is used here. Secondly [15], considers only full cache locking while we consider partial cache locking, trying to take advantage from both cache content locking and localities in cache accesses. Lastly, the content selection algorithm used here tries to select the most *significant* basic blocks rather than memory blocks to cooperate with BBIP.

## BBIP Mechanism

In this section, we will describe how to perform instruction prefetching at the basic block level in the BBIP mechanism.

### Mechanism description

In BBIP, all memory blocks of the basic block where the instruction lies are loaded into the instruction cache when an instruction access misses. As the size is not constant for different basic blocks, additional information of the basic blocks must be kept in the system in advance to direct the prefetching. This does not make sense in general-purpose systems, where the set of the applications varies from time to time, and new applications may be added to the system without any priori knowledge. On the contrary, the tasks running in real-time systems, especially hard real-time systems, are usually fixed and pre-defined when the system is designed. And thus the program behavior data of the task set are available to direct the system design, such as the cache architecture design. Therefore, it is possible to collect the information of all basic blocks in the real-time applications with static program analysis technologies and use it to direct the instruction prefetching.

In the following part, we will describe how to implement the BBIP mechanism in hardware and software.

### Hardware implementation

To support the BBIP mechanism, information of the basic blocks existing in the programs must be kept in a hardware structure to direct the prefetching. It is feasible in real-time system due to the fact that the basic blocks existing in real-time applications are often very limited. For example, most of the benchmarks we used in our evaluation have a size of no more than 32 static instructions, typically less than 16 instructions and occupy two or three cache blocks (as shown in Table 1). As the prefetching is performed only for the basic block which occupies more than one cache block, no information is kept for those whose size is smaller or equal to the cache block size. So less than 64 basic blocks have to be recorded for benchmarks in Table 1 to direct the prefetching, except *adpcm*, for which about 100 items are recorded.

**Table 1:** Distributions of the size of the basic blocks in the benchmarks.

NOI	[1,2]	[3–4]	[5–8]	[9–16]	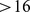	Total BBs
adpcm	49	18	38	28	10	143
cnt	6	8	7	0	1	22
crc	12	6	2	7	1	28
edn	14	8	11	13	7	53
fft1	15	21	10	9	2	57
fir	9	4	0	4	0	17
lms	20	6	15	16	1	58
matmult	6	5	8	2	1	22
qurt	13	3	5	7	1	29
Total	144	79	96	86	24	429
Percentage	33.6%	18.4%	22.4%	20.0%	5.6%	100%

In our design, a table called *basic block information table* (BBIT) is maintained to record information of basic blocks in the active program to direct the prefetching. Table 2 gives an example of a BBIT containing 100 items.

**Table 2:** Example of a BBIT with 100 items.

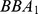	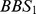
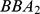	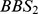
…	…
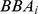	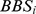
…	…
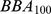	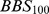

Each item in BBIT contains both the start address of the basic block aligned to cache block (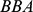), and the size of the basic block in cache block size (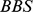). For example, for a 48-byte long basic block with the first instruction at 0x400128, which runs on a system with an instruction cache with 32-byte blocks, the 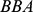 and 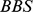 are 0x400120 and 2, respectively.

Suppose a program runs on a system with 4-byte instruction length and a 4K-byte instruction cache with 64-byte blocks, the first 26 bits of the address records the block address (*tag*+*set*), and the remaining 6 bits act as offset to index into the cache block. A number occupying 6 binary bits is able to record a number as large as 63, which is enough to record 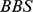. Therefore, one word (4-byte) is enough to record 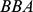 and 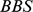 as one item in BBIT, and a BBIT with 100 items will occupy 400 bytes.

**Figure 1:**
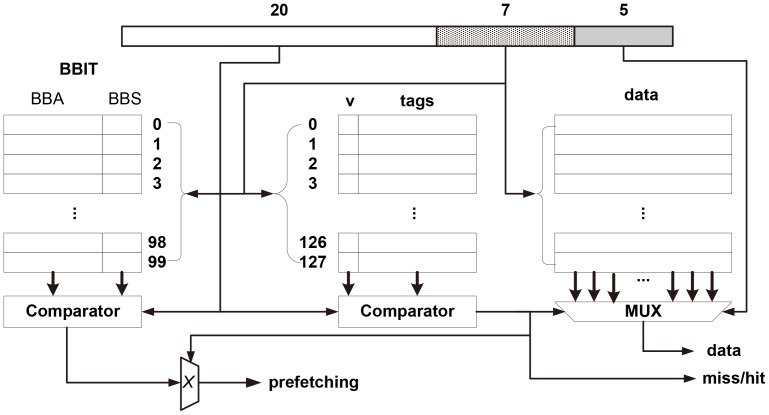
Example of a 4k-byte instruction cache with BBIP equipped. The cache is directly mapped and has 32-byte blocks. And the BBIT has 100 items.

The access to an instruction cache with BBIP mechanism (as depicted in Figure 1) is divided into the following steps:

1. **Dispatching**-Every time an instruction access is issued and dispatched to the instruction cache, the address is divided into three segments: *tag*, *set* and *offset*, according to the cache configuration.
2. **Lookup**-Compared to traditional cache structure, cache with BBIP will perform BBIT lookup while searching the tag table. So the step is further divided into two sub-steps performed concurrently:
**Tag Lookup**-The *set* part of the address is used to index into the tag table to find the target set where the address lies, and then the *tag* part is compared with those of the blocks in the target set. If there is a hit, the corresponding block in the cache is fetched or updated, depending on a read or write request. Otherwise, the lookup fails.
**BBIT Lookup**-The 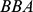
s of the items in BBIT are compared with the *block address* composed of the *tag* and *set* parts of the address. If it matches, the 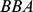 and 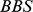 are returned, otherwise invalid 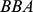 and 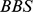 are returned to indicate no prefetching needed.
3. **Prefetching**-If the *Tag Lookup* step fails and the *BBIT Lookup* returns valid 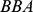 and 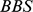 values, all blocks indicated by 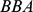 and 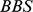 are fetched into the cache and the blocks selected by the replacement policy are swapped out; otherwise, no prefetching is performed.

As prefetching only occurs when an instruction access misses, which will then trigger a memory access (termed as *slow path*) and occurs uncommonly, less than 1 in 10 instruction accesses in our evaluation (see Table 3), the prefetching operation will not be too often. Also, the size of most basic blocks is often small (e.g. covering 2 or 3 memory blocks) as shown in Table 1, so the prefetching operation can, if not totally, mostly overlap with the executions of the instructions of the memory blocks of the same basic block which are already prefetched in a non-blocking instruction cache. Also, the prefetching overhead can be reduced in a memory system in burst mode. Moreover, if the items in BBIT are sorted and then maybe binary search is possible to accelerate the lookup for a given 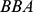 value. A more reasonable implementation of BBIT is to construct a hardware structure which works as a cache (called *BBIT cache*). In the BBIT cache, the 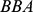
s act as tags (termed as *BBA Tag*) and the data buffer stores 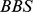. Every time a BBIT lookup is issued, the 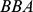 of the basic block is used as the key to hash in the BBIT cache and locate the item where the basic block is mapped. If there is a hit, the 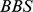 value is fetched and both 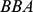 and 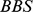 flow to the prefetch unit to direct the prefetching. To avoid the potential conflict in the 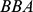, some features as that existing in traditional cache, such as using a set-associative cache, may be needed. All these details are out in the scope of the paper and thus no details are given here.

**Table 3:** Miss-rates collected in a simulation where 4-way 512-byte cache with 32-byte blocks is used.

program	accesses	misses	miss rate
adpcm	124977	562	0.45%
cnt	4071	25	0.61%
crc	21464	34	0.16%
edn	63756	816	01.28%
fft1	1511	44	02.91%
fir	251090	18	00.01%
lms	219199	7405	03.38%
matmult	133668	29	00.02%
qurt	638	58	09.09%

It is noted that, as the BBIP mechanism only serves as a feature attached to the normal cache structure (as shown in Figure 1), it is possible for a cache with the BBIP feature to act without instruction prefetching by disabling the *BBIT Lookup*. This feature makes BBIP still work in a dilemma. For example, in a multi-task real-time system where some task has a basic block set too large to be kept in the BBIT, the BBIP feature can then be disabled temporally when the task is swapped in and later re-enabled once it is swapped out. This also implies that we can provide differentiated cache performance to meet other requirements, such as tightness of WCET analysis or power consumption. Moreover, to limit the hardware cost to a certain level, we can fix the size of BBIT (for example 32 items) and load the most *significant* basic blocks selected by some selection algorithms to provide sub-optimal performance.

### Software and hardware combined implementation

Apart from being implemented in hardware, the BBIP mechanism can also be implemented in software with the aid of the hardware features of modern processors.

Modern microprocessors often provide some non-blocking software prefetching instructions, which prefetches some cache line(s) (determined by some prefetching algorithms) in advance to avoid the following instruction access misses. These instructions often use some bits to record information, such as prefetching distance, to direct the prefetching. Most basic blocks in real-time applications are small, usually no more than 16 memory blocks. Therefore, several bits (such as 4) will be sufficient to record the length of a basic block in order to direct the prefetching. An alternative is available on some processors. For example, in ISAs like PISA in SimpleScalar where each instruction is 8-byte long, certain fields of the instructions are unused or reserved for future extensions. If this is true, it is possible to make use of these fields to encode the prefetching information (termed as PIF) without inserting extra instructions. Every time the execution goes to a basic block other than the one just executed, the first instruction, which has the prefetching information encoded in its instruction word, is fetched and decoded. If a cache miss occurs and the PIF indicates a prefetching is possible, all the following memory blocks are prefetched into the instruction cache. For basic blocks whose size is too large to record in the reserved field, just leave them un-recorded and no prefetching is performed.

With the support of instruction prefetching in the ISA, the compiler's job is not too complicated. Firstly, it performs program path analysis on the program to collect all basic blocks, including start address and size (in memory block). The process may need underlying hardware information, such as the cache configuration of the target platform. Then, it performs static cache analysis (such as [16]) to classify every access to the first instruction of each basic block as either *always-miss*, *always-hit*, *first-miss* and *first-hit*. After that, the compiler replaces the first instruction with a call to a sequence of instructions that perform prefetching before executing the instruction at the entry of each *always-miss* basic blocks or encodes the prefetching information into the first instruction of the basic block. As the prefetching instructions are only inserted inside a basic block, the execution path will not be affected. Also, to avoid any side effect the prefetching routine may bring into the instruction cache, it can be placed in a memory section which bypasses the instruction cache when accessed. Moreover, as BBIP improves WCET, and the prefetching instructions will always be executed once the execution enters the basic block, the program will benefit from the prefetching.

## Methods: BBIP-Combined Partial Instruction Cache Locking

Embedded processors are often equipped with relative small caches and provide a locking mechanism to provide timing predictability. The cache locking feature is available in many commercial processors, such as Motorola ColdFire MCF 5249, PowerPC 603e and ARM 940T. In this paper, way locking is chosen to implement the partial locking mechanism as it is easier to evaluate and more intuitive to show to what degree the cache is locked to achieve the best WCET improvement. It is noted that, line locking can also apply to the partial cache locking and provide at least as good performance due to its fine-grained control over the cache contents. In our partial cache locking methods, the unlocked portion acts as a normal cache with the BBIP feature equipped.

### Overview

In our BBIP-combined partial cache locking method, for a *n-way* cache with *k* ways locked, the unlocked portion acts just as a set-associative cache with *n-k* ways, where the BBIP is employed.

We only consider static cache locking in this paper, that is, the cache contents selected by the content selecting algorithm discussed below are loaded and locked in the instruction cache before the execution of the program and kept unchanged until end of the execution. Some additional code for cache locking must be executed before the program starting execution. One option is inserting some code at the beginning of the original code and executing them to load selected cache contents, which will change the code memory layout and in turn change the mapping of instructions to the cache sets. To avoid this problem [4], uses the trampolines approach proposed in [17], in which codes to load and lock the cache contents in the cache are inserted at the end of the program as a trampoline. And a dummy NOP instruction at the entry point of the program is replaced by a call to the trampoline.

In a partially locked cache, every access to the instruction cache may lead to one of the following three results: hits in the locked ways (termed as HLW), hits in the unlocked way or misses. If the hit occurs in the locked ways, the instruction is returned and no other operations are performed; if it hits in the unlocked way, the hit block is switched to the most recently used (MRU) way. While if the access misses, the least recently used (LRU) cache line in the unlocked portion is selected to swap out and a memory block is swapped in and the access is served. Unlike existing locking mechanism implemented at the memory block level, the partial locking proposed here is performed at the basic block level, which means that if one memory block is selected to be locked, other blocks in the same basic block will also be locked. And in caches with BBIP, the miss may only occur at the first memory block of each access to a basic block. It is now clear that if a miss occurs in our partial locking cache and the basic block where the instruction lies spans more than one memory block, all the following memory blocks are prefetched into the unlocked portion of the cache to serve following accesses to the instructions in the same basic block.

As the unlocked portion of the cache acts just as a set-associative cache, the work flow of the prefetching will follow exactly the classic way performed in a normal cache with the BBIP mechanism described above. Here, we will omit it in our description and focus on how to select the most *significant* basic blocks to lock in the cache in order to maximize the worst-case cache performance and improve the WCET.

### Two-phase cache content selection algorithm

The content selection algorithms existing are almost proposed to select cache contents at the memory block level. This does not work in our partial cache locking context. The reason is intuitive. In memory block based cache content selection algorithm, the selecting of memory blocks is performed independently for different sets. So only memory blocks mapped to set *i* are evaluated to select to lock in set *i*, and memory blocks mapped to set *j* (i
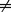
j) has no impact on the decision of the content selection of set *i*. The BBIP mechanism will be messed up when some memory blocks of a basic block are chosen to lock while the others are left unlocked and BBIP is performed on them. To take advantage of both BBIP and partial cache locking, we proposed a content selection algorithm performed at the basic block level. The main difference of our algorithm is that, when considering to lock a memory block in the cache, all memory blocks in the same basic block are considered and the *average* value is used as the metric to make the selection decision. Once a locking decision is made, all the memory blocks in the basic block are locked, and the next selection begins at the set after the last cache block covered by the basic block.

It is easy to deduce that selecting the most *significant* basic blocks to lock to minimize the WCET is NP-hard. Considering a special case where all basic blocks have the same size, one memory block, the problem is equivalent to the 0/1 knapsack problem, which is NP-hard. In this paper, we proposed a two-phase reference-based algorithm to greedily select the most *significant* basic blocks to lock in the cache, the idea behind which is quite similar to that used in [6]. The difference is that, we lock the most frequently referenced basic blocks instead of memory lines in the cache. The algorithm works as follows:

• Profiling.

Control flow graph (CFG) analysis is performed to collect the basic blocks existing in the program, where the start address and size (in block) of each basic block are recorded. Then static WCET analysis (see [18]) is performed, which returns the execution count of each basic block on the worst-case execution path.

• Content Selection.

Based on the information collected in the *Profiling* phase, we use a greedy algorithm to select the most *significant* basic blocks to lock in the cache. The greedy content selection algorithm is detailed in the following paragraph.

**Figure 2:**
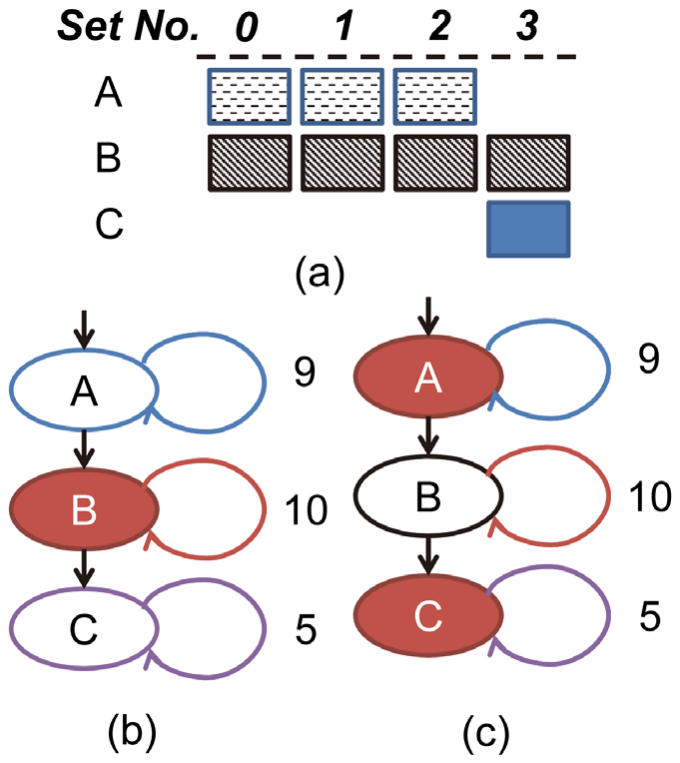
Example of a *bad* (b) and *good* (c) content selection, where the red nodes are locked.

The reference count is used as the metric to evaluate the profit a basic block may bring if locked in the cache. However, locking the most frequently referenced basic blocks does not necessarily bring the biggest profit. We can explain it with a simple example, as shown in Figure 2. Suppose a program runs on a processor with direct-mapped cache which has 4 sets and the worst-case execution path of the program includes three basic blocks A, B and C (see Figure 2 (b) and (c)). The basic blocks A, B and C occupy 3, 4 and 1 memory blocks respectively and their mapping to the cache sets as shown in Figure 2 (a). Moreover, based on the static analysis, the basic blocks A, B and C are executed 9, 10 and 5 times, respectively. If we select the basic blocks with the biggest reference count to lock in the cache, the basic block B is selected (see Figure 2 (b)) and 10 cache misses are eliminated. However, if the basic block A and C are locked, 14 misses are avoided.

In this paper, we use reference per memory block (RPMB) as the metric to select the most *significant* basic blocks to lock. The RPMB of a basic block *B* is defined as
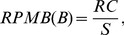
(1)where RC is the reference count of *B* and S indicates the size of *B* in memory block. For the basic blocks A, B and C in Figure 2, the RPMBs are 3, 2.5 and 5, respectively. So the basic block C and A are selected.

To lock the basic blocks with the biggest RPMB in cache, the following operations are performed:

1. Categorizing.

All the basic blocks collected in the *Profiling* phase are categorized into different sets (called *available-to-select* sets) according to their start addresses. For a basic block whose start address is mapped to set *i* (termed 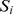), it goes to set *i* (termed 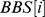). So for a cache with *n* sets, at most *n available-to-select* sets, 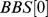, 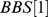,…, 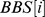,…, 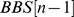, are created. Moreover, all the basic blocks whose size is bigger than the set number of the cache are discarded as they cannot be locked in the cache as a whole.

2. Sorting.

The basic blocks in each *available-to-select* set created in the *Categorizing* step are sorted with RPMB as the primary key and the size of the basic block as the secondary key. That is, the basic blocks are ordered degressively according to the RPMB of each basic block, and for those with identical RPMB, the basic blocks with bigger size are placed before the ones with smaller size. Further, if two basic blocks in the same set have the same size and reference count, their order is random. After the operation, at most *n* ordered sets (represented as OBBS [0: n-1] hereafter) are generated and passed to the following operation step. It is noted that, although we call OBBS[i] (where 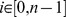) a set, it is ordered and organized as a linked list.

3. Selecting.

**Figure 3:**
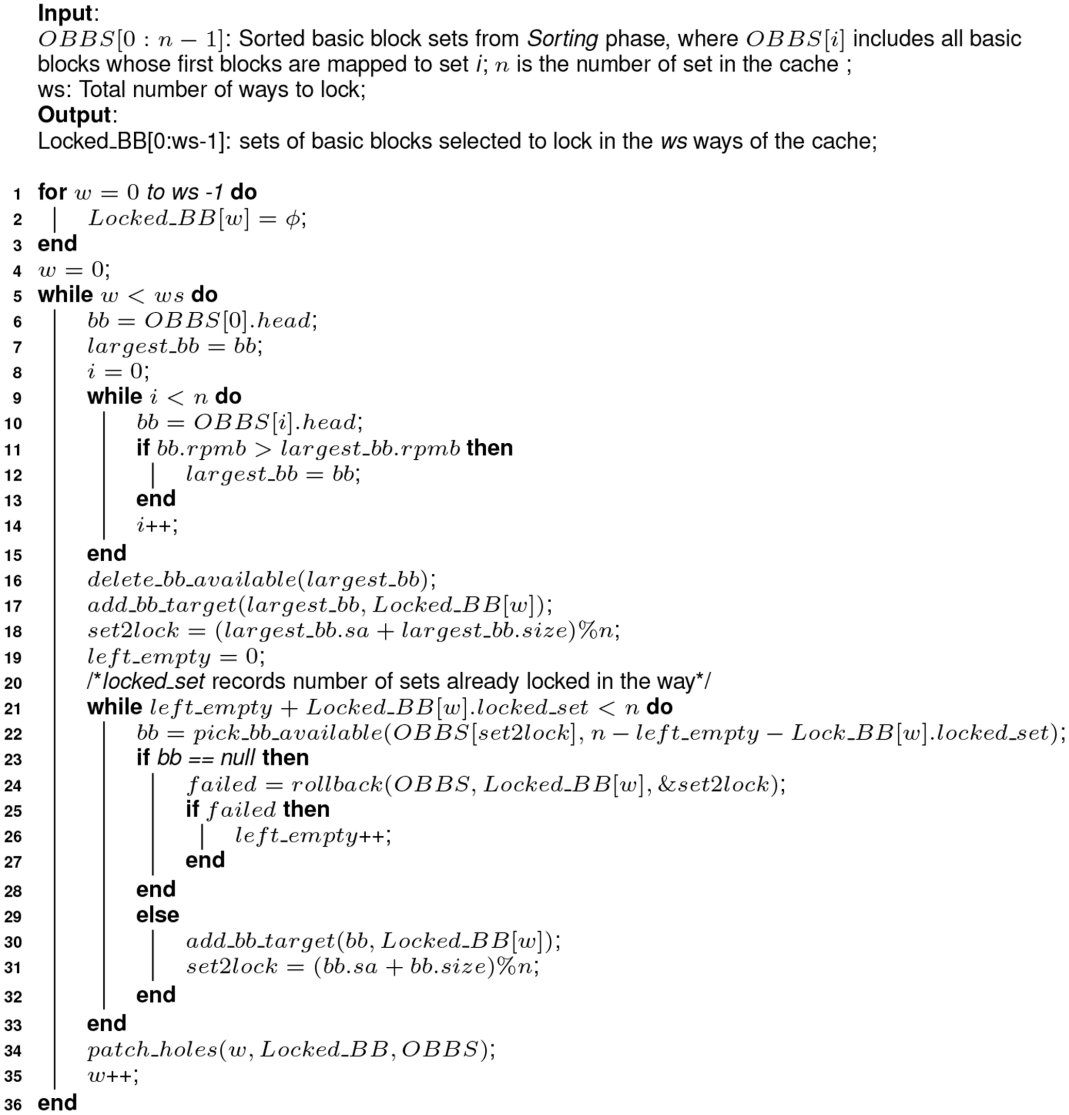
Algorithm 1: Greedy partial cache content selection algorithm.

The selection of contents to lock is performed independently for each way. That is, if there are k ways (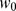, 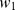,…, 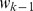) to lock, the basic blocks from OBBS[0:n-1] are selected to lock in all sets of 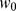, then 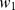, and finally 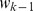 (see Figure 3).

4. Rollback.

In the *Selecting* phase, the basic blocks with the largest RPMB are selected, and if the selected basic blocks cover all sets in a given cache way, the content selection operation of the way will terminate without further process. However, although occurring rarely, sometimes it is just impossible to select a qualified basic block to fill in the set under consideration. Let's consider the following two cases:

- *null-set* case: the *available-to-select* set OBBS[i] is empty while trying to select a basic block starting from set *i*.
- *size-conflict* case: each basic block in OBBS[i] covers more blocks than that left unlocked in the way under consideration, when considering the locking of the block in set *i*.

**Figure 4:**
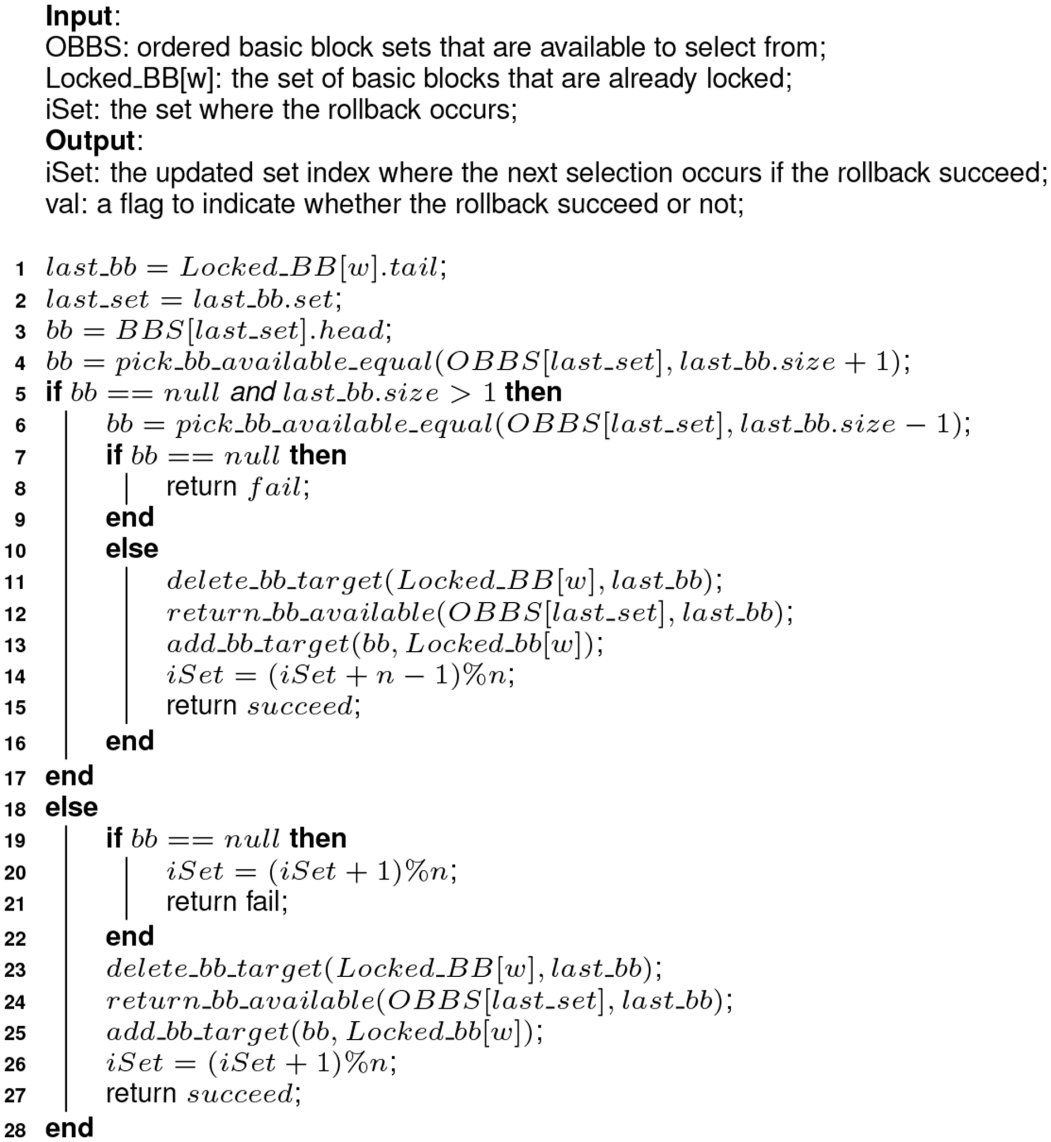
Algorithm 2: Implementation of the one-step roll-back function–rollback().

To handle these unusual cases in the *Selecting* phase, we add an additional step to patch, termed as *one-step rollback* (see Figure 4).

5. Patching.

**Figure 5:**
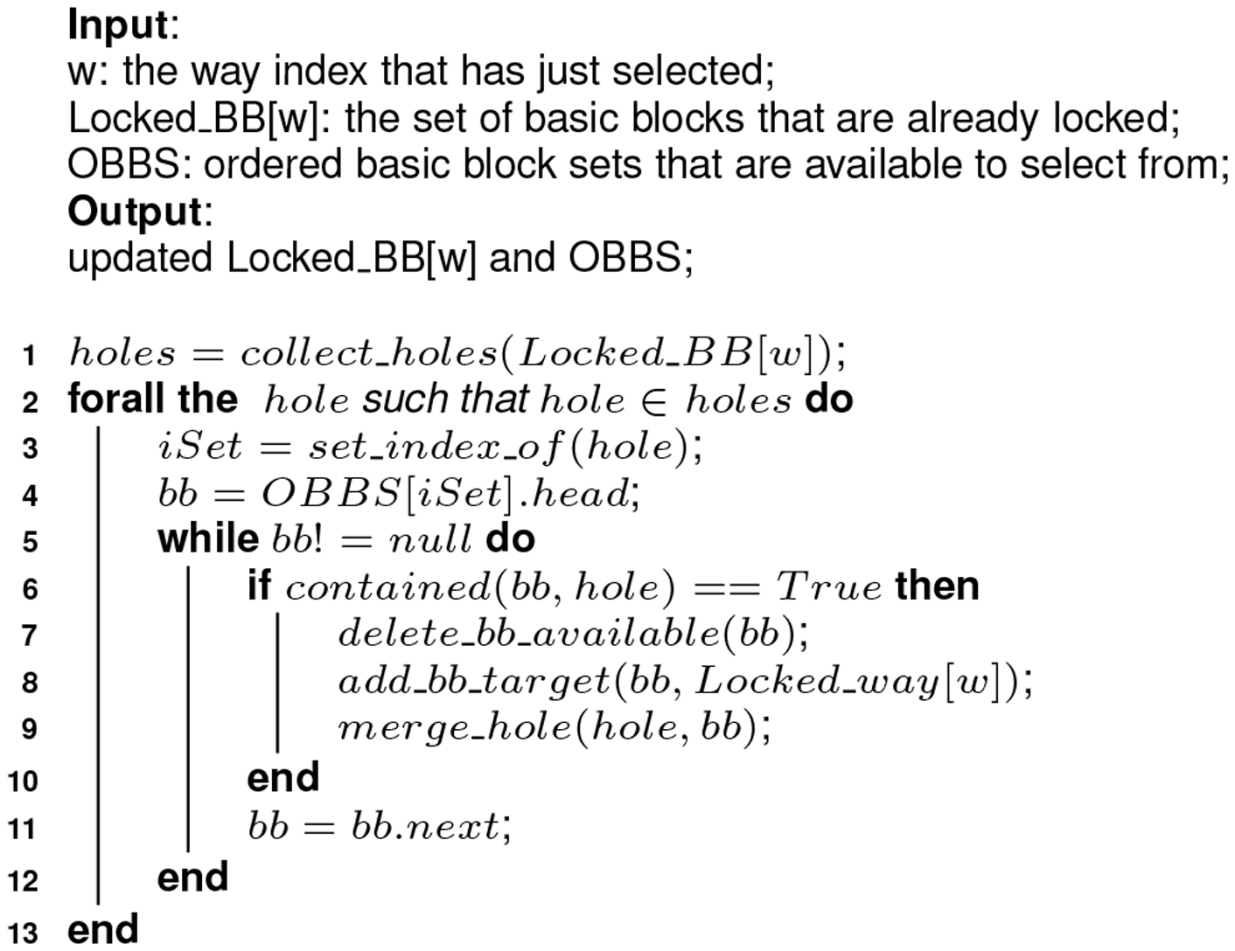
Algorithm 3: Implementation of patch_holes().

After all the above steps, almost all bytes in a way are locked, but there may also be some *holes* between the selected basic blocks. A hole is defined as the bytes between two adjacent basic blocks selected to lock. For example, in a 512-byte cache with 32-byte blocks, the basic blocks *A* and *B* range from byte 0 of set 0 to byte 15 of set 1, and byte 0 of set 2 to byte 31 of set 2, respectively. Then there is a hole between *A* and *B* ranging from byte 16 to byte 31 in set 1. As instructions are fetched into the cache block by block, the instructions located in the *hole* between *A* and *B* are also brought in when *A* is selected to be locked. So if a basic block *C* fits in the *hole*, it should also be labeled as being *locked*. The *selecting* and *rollback* phases do not consider this kind of *side effect*, and an extra function called *patch_holes* is defined (see Figure 5) to handle it. For a cache with *ns* sets, there are at most *ns* holes to patch after the *selecting* and *rollback* phases.

#### Algorithm description

In Figure 3, the first three lines (from line 1 to 3) initialize the locked basic block sets with nothing locked in. From line 4 to 36, the cache content selection is performed way by way at the basic block level until all ways predefined are locked, according to the greedy content selection algorithm. Line 6 to 15 tries to locate the basic block with the largest RPMB by traversing the heads of all *n available-to-select* sets, from OBBS[0] to OBBS[n-1]. Line 16 unlinks the located basic block from the *available-to-select* set where it is linked. In line 17, the located basic block is added to the target set, which keeps all basic blocks selected to lock in the cache way under consideration. The target set for each way is organized as a linked list, with the first basic block selected as the head and the most recently selected one as the tail. The variable *set2lock* records the next set to fill and it is set in line 18. Line 19 initializes the 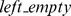 variable to zero, which records the times of rollback failure. Every time the rollback operation fails, the cache block of way 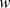 in set 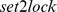 is skipped and left unlocked. From line 21 to 33, all sets of the way 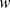 are considered to lock some basic blocks selected from 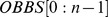. Line 22 assigns the first qualified basic block to *bb* by calling 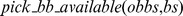, which tries to find the first basic blocks with a size no bigger than *bs* from the ordered basic block set *obbs*. Line 23 to 28 handles the uncommon cases in the *Selecting* phase when no qualified basic block is available to lock in set *set2lock* and the *rollback* operation is performed as a remedy. Otherwise, line 29 to 32 are executed to add the selected basic block to the target set and update the *set2lock* variable. In line 24, the *rollback* function is called and the variable *set2lock* is updated during the invoking. If the *rollback* operation fails, the variable *left_empty* is updated as figured out from line 25 to 27. Otherwise, the selection continues at the updated set *set2lock*. In line 34, a function to patch the holes existing between the selected basic blocks in the current way is called. The implementation is described in Figure 5.

Figure 4 depicts the process of the *rollback* function called in Figure 3 (at line 23). Line 1 locates the most recently selected basic block and line 2 gets the set the first block of the basic block mapped to. Line 3 tries to pick a basic block whose size is exactly one memory block larger than the one just selected. If a qualified basic block is found, unlink it from the 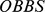 and assign it to *bb*. If a valid *bb* is found, the execution goes to line 22. The basic block located in line 1 is deleted from the target set (line 22) and returned to the ordered *available-to-select* set (line 23). Line 24 adds *bb* to the target set and line 25 updates the variable denoting the next set to lock. And the function returns a flag to inform the caller the *rollback* operation succeeds (line 26). On the other hand, if the re-selection of basic block in line 3 failed and the size of the basic block got in line 1 is bigger than one memory block, the execution goes to the other branch, from line 4 to 16. Line 5 tries to pick another basic block from the same 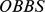 as that in line 1. The basic block should have a size one memory block smaller. If the operation failed again, the one-step rollback operation fails and the function returns, shown from line 6 to 8. Otherwise, similar operations are performed from line 10 to 14 as that from line 22 to 26. Codes from line 18 to 21 handle the case where line 3 returns no valid basic block and the last selected basic block covers one memory block. In this case, we just update the set index where the next selection occurs and return a flag to indicate the failure of the rollback operation.

It is noted that, any operations on 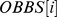, where 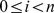, should follow the rule that, the order of 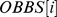 (described as a linked list) should be kept. That is, if a basic block is returned to the list, it should be inserted in the space where the basic block previous has a bigger RPMB and the one after it has a smaller RPMB. Moreover, we only select the basic blocks whose size is one memory blocks longer or shorter than the one just being selected as candidates to roll back. There is a balance here. On the one hand, we expect as few blocks as possible are skipped once the *rollback* operation fails. One the other hand, the *rollbakc* operation is designed to be not too complicated to avoid deadlock.

Figure 5 describes the operations to patch the holes that may exist in the selected basic blocks in the current way. Line 1 collects holes that may exist between the basic blocks that have been selected in the way. For a cache with *n* sets, there are at most *n* holes. From line 2 to 13, the basic blocks in the *available-to-select* sets that can fit in the holes are selected and added to the target set. Line 3 returns the set where the hole begins. From line 4 to 12, the *avail-to-select* set starting at the same set as the hole are traversed to find if a valid basic block exists. The function *contained* in line 6 is used to check if a basic block can be filled in the hole or not. If it can, the function returns true, otherwise false. If a valid basic block is found, it is deleted from the *available-to-select* set (line 7) and added to the target set (line 8). After a hole is patched, new hole(s), at most 2, may appear, as the fragments in memory allocation in the Linux kernel. The function *merge_hole* (in line 9) is used to handle the *fragmented* holes after one patching of a hole. The idea is very similar to the one that behinds the memory management mechanism in the Linux kernel. Here, we just ignore the details.

#### Complexity

**Figure 6:**
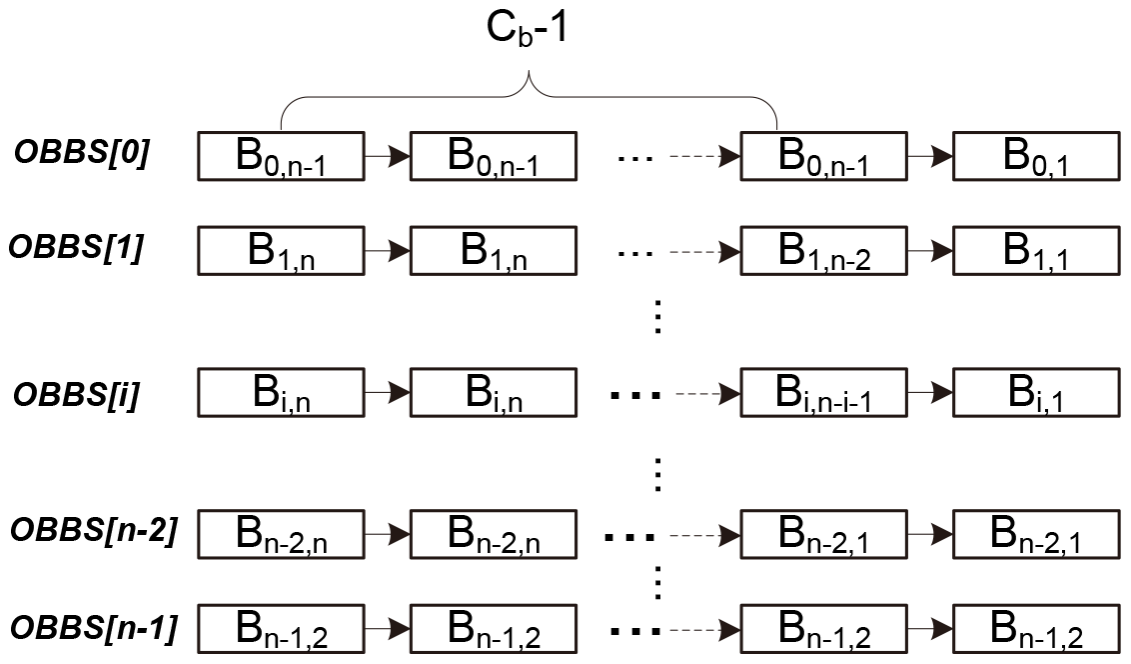
Example of the worst-case cost of Algorithm 1 as shown in Figure 3.

Suppose that there are 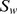 ways to lock in Algorithm 1 in Figure 3, the cache has *n* sets and the *available-to-select* set (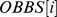) for each cache set includes at most 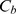 basic blocks. The loop from line 9 to 15 will iterate 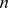 times, and the cost of line 16 to 19 is 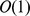, so the cost of Algorithm 1 in Figure 3 is determined by the cost of the loop from line 21 to 33 and the *patching* step at line 34. It is easy to deduce that, the best-case and worst-case cost of *patch_holes* is zero and 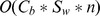, respectively. The best-case cost of the loop is 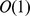, when the basic block with biggest RPMB covers exactly all sets in the way and thus no traversing of the *available-to-select* and rollback operations are needed. In this case, the cost of Algorithm 1 in Figure 3 is 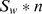. The worst-case cost of the *pick_bb_available* function at line 22 is 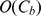. And at most 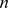 calls to *rollback* function are needed. As the *rollback* operation is limited to one step, no avalanche effect exists in Algorithm 2 as shown in Figure 4. It is easy to deduce that the cost of Algorithm 2 is 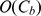. The example in Figure 6 shows a scene where the worst-case cost is achieved. In Figure 6, 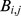 represents a basic block in 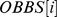 (with 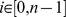), where 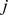 (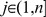) is the basic block size in memory block. So, 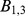 is a basic block in 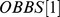, mapped to set 1, 2 and 3 (if 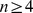). We also assume that the head of 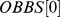 have the biggest RPMB value among all basic blocks in the program. Then the selection begins at set *0*. Table 4 shows the process to lock one way for the scene in Figure 6, where S and R denotes the *selecting* and *rollback* operation, respectively. Under our assumption above, after 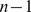 comparisons (from line 9 to 15), the head of 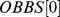 (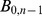) is selected and added to the target set. As its size is 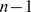, 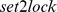 is assigned to 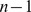, which means the next selecting occurs in 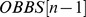. The function 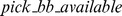 traverses 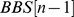 from head to tail ( with 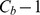 comparisons) to find a basic block whose size is 1, and no qualified basic block is found. Then the *rollback* operation is performed and 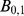 at the tail of 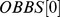 is selected after 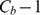 comparisons. At this point, the content selection at set 0 completes and the selection process goes to set 1, where the similar thing happens. From Table 4, it is clear that the cost to select a *proper* basic block is 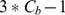 for one set other than set 0, which is 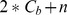. So the total cost to lock one way is 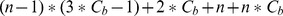, which is 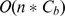. Therefore, the worst-case cost to lock 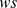 way is 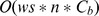.

**Table 4:** The basic block selection process to lock one way in the scene presented in Figure 6.

Step	Selected_BB	MB locked	Action	Complexity of Selection	Complexity of rollback
1	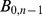	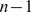	S	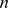	0
2	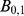	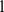	S,R	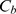	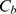
3	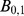 , 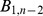	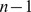	S	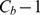	0
4	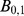 , 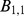	2	S,R	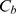	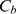
…	…	…	…	…	…
2i-1	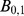 ,, 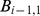 , 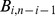	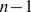	S	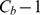	0
2i	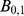 ,, 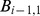 , 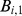	1	S,R	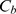	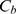
…	…	…	…	…	…
2n-1	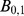 , 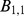 ,, 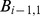 ,, 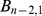	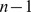	S	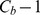	0
2n	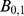 , 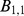 ,, 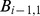 ,, 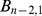	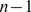	S,R	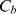	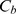

It is noted that, although we do not detail the mechanism to detect and avoid deadline during the content selection in Algorithm 1 and 2 as shown in Figure 3 and 4, it is indispensable to handle the cases like the two steps in Table 4.

#### Safety of the estimated WCET

Locking some contents in the cache may cause the worst-case execution path (WCEP) switching from one to another. Sticking to the original WCEP may make an underestimation of the real WCET. That is, the estimated WCET may be unsafe. The Chronos framework makes several contributions to handle this. Firstly, it calculates the WCET of a basic block in all possible execution contexts by considering the timing effect of its prologues and epilogues. The prologue and epilogue are a piece of instructions before and after the basic block under consideration that affect its timing and constitute its context. They are collected with the aid of the execution graph of the basic block, which captures both data dependencies and resource contentions. Once the estimation of the WCETs of all the basic blocks is completed, an Integer Linear Program (ILP) solver is used to produce the program's WCET estimation. The objective function of the ILP problem looks like
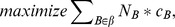
where 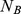 is an ILP variable denoting the execution count of basic block 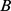 and 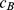 is a constant denoting the estimated WCET of 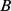. And 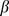 is the set of basic blocks of the program. The linear constraints on 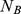 are derived from the control flow graphic and pipeline analysis. It is clear that, locking a basic block 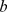 in the cache will change its 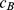 and also affects the pipeline analysis, both of which affect 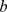
's execution count and contribution to the final WCET estimate. This implies, a *hot* basic block selected originally to be locked may become *cold* and contribute little to the WCET estimate improvement. Our content selection algorithm is not optimized to improve this at this moment. It is noted that, although the WCET estimates presented here may be sub-optimal in some cases, but they are safe. This is because, the WCET of every basic block of the program is re-estimated and the ILP formation covers all basic blocks and their execution contexts as well as the affected cache and pipeline states. That is to say, the Chronos framework can cover the changing of WCEP and always provides a safe WCET estimation. Also, it implies that the instruction prefetching combined partial cache locking mechanism may provide more remarkable improvement if the content selection algorithm is improved. For more information about the idea of the WCET estimation used in Chronos, please refer to [18].

## Experiments and Results

In this section, we present the experimental evaluation of our BBIP-combined partial cache locking mechanism in a uni-processor context. The performance is evaluated by comparing its WCET improvement over the static analysis method used by Li et al. in [19] and full cache locking used by Falk et al. in [8]. We select the full cache locking method to compare with based on the following two aspects. Firstly, it should be designed for single task, which excludes a lot of work targeting multitasking system, such as [6], [15], [20] and [21]. Secondly, it should not use other optimization technologies (such as code layout/placement) to aid the full cache locking method, such as [9]. In [9], the authors assume that any memory block can be locked in any cache set, while in our locking method, we do consider the cache mapping function in the locking algorithm. To achieve a fair comparison, we choose basic blocks as locking granularity instead of procedures originally used by Falk et al. in [8].

### Assumptions

In the evaluation, we assume only one level of instruction cache. That is, an instruction access is either cache hit or leads to one access to the main memory. We also assume a perfect data cache, and all data accesses will hit in the data cache. When the cache is fully locked, the first access to an unlocked block will miss and be fetched into a *buffer* whose size is exactly one cache line. The following accesses in the same block will hit in the buffer, which are equivalent to cache hits. We use burst mode in the memory system as that in Simplescalar, and the latencies of the first and following chunk are given in Table 5. Also, we use a hardware implementation of the BBIP mechanism, and all the basic blocks that can be totally filled in the cache are stored in BBIT.

**Table 5:** Configurations of the evaluation framework.

Cache size	512B, 1KB
Cache block size	16B, 32B, 64B
Associativity	4-way
Replace Policy	LRU
Cache hit latency	1-cycle
First chunk latency	30 cycles
Following chunk latency	2 cycles
Memory bus width	8-byte
Branch prediction	perfect
Pipeline	in-order

### Evaluation methodology

We modify the evaluation framework developed in our previous work [5] to implement partial and full cache locking. The model used in [5] is built on top of the open-source WCET tool-Chronos [19] to equip the BBIP feature in the instruction cache and enclose it in the static analysis. We use 9 programs from SNU real-time benchmarks [22] (as shown in Table 6) for evaluation. In Table 6, *Bytes*, *LOC*, and *NOI* represent the size of the source code file, the number of lines of the source code, and the number of instructions in the binary code, respectively. All the benchmarks are compiled to SimpleScalar PISA instruction set [23] — a MIPS-like ISA — with gcc cross-compiler. The compiler options are -O2 and -g. Every instruction in PISA is 8-byte long.

**Table 6:** Characteristics of the selected SNU real-time benchmarks.

Benchmark	Description	Bytes	LOC	NOI
adpcm	Adaptive pulse code modulation algorithm	26852	879	928
cnt	Counts non-negative numbers in a matrix	2880	267	106
crc	Cyclic redundancy check computation on 40 bytes of data	5168	128	150
edn	Finite Impulse Response (FIR) filter calculations.	10563	285	550
fft1	1024-point Fast Fourier Transform using the Cooly-Turkey algorithm	6244	219	312
fir	Finite impulse response filter (signal processing algorithms) over a 700 items long sample.	11965	276	74
lms	LMS adaptive signal enhancement. The input signal is a sine wave with added white noise	7720	261	342
matmult	Matrix multiplication of two 20×20 matrices	3737	163	122
qurt	Root computation of quadratic equations	4898	166	170

To focus on the impact of instruction cache on the WCET analysis of real-time applications, we disable the dynamic branch prediction and out-of-order pipeline model provided in Chronos. More detailed configurations of the framework are listed in Table 5.

To make the discussion clearer, we define the following framework configurations.

- **Perfect-ICache**: We use a perfect instruction cache as the baseline to normalize all the estimated WCET values in the other configurations below. In this configuration, neither mandatory misses nor conflict misses exist and all instruction accesses hit in the L1 instruction cache. Thus, the latency is always one cycle for all instruction accesses.
- **NP-NL**: The WCET value is estimated on an instruction cache where no BBIP and no cache locking is performed. The configuration can also be referred as *static analysis*.
- **NP-PL**: The partial cache locking is performed while no BBIP is used for the instruction cache. The two-phase cache content selection algorithm is used to select the basic blocks locked in the instruction cache.
- **NP-FL**: The full cache locking is performed while no BBIP is performed for the instruction cache. The I-Cache content selection algorithm used in [8] is used for cache content selection.
- **P-NL**: The instruction cache is not locked and BBIP is enabled to perform instruction prefetching.
- **P-PL**: The BBIP mechanism is enabled and partial cache locking is performed for the instruction cache. The same cache content selection algorithm as that used in NP-PL is adopted.
- **P-FL**: The BBIP mechanism is enabled but actually does nothing, and all ways of the instruction cache are locked. The configuration is actually identical to NP-FL, so in following discussions we omit it.

In the evaluation, we collect the estimated WCET values of all the benchmarks listed in Table 6 for the above configurations. And then we use WCET values got in Perfect-ICache to normalize the estimated WCET values collected in the other five configurations. In the Perfect-ICache configuration, all instruction accesses hit in the instruction cache and thus lead to the lowest estimated WCET values of the six configurations. In other words, all normalized WCET values are above 1. The lower the value is, the better the configuration works in improving WCET. In the following discussions, if the WCET estimate collected in a configuration is very close (for example less than 10% larger) to that in the Perfect-ICache, we say the WCET estimate is *perfect*.

### Discussions of overheads of cache content locking and BBIP

To lock some contents in the cache, typically two steps should be performed. Firstly, the contents are loaded into the cache from the main memory by issuing read operations to the first instruction of each memory block with *load* instruction. Secondly, a piece of instructions are executed to perform cache locking. In the processors with instruction cache locking feature, several assemble instructions is enough to lock some ways of the instruction cache [24].

In this paper, the instruction cache is locked statically, which means some contents are selected and locked in the cache at the start-up phase of the system and kept unchanged during the execution of the program. So the time used to lock some contents in the cache should not be included in the WCET estimation of the program in a mono-task context. In the following discussion, the WCET estimates related to the partial and full cache locking mechanism excludes the latencies used to perform cache locking.

When BBIP mechanism is enabled, it will incur some overheads to prefetch the following memory block(s). In the evaluation, we use a simple way to model the overhead. The penalty to load the first memory block of a missed basic block equals a cache block miss penalty, that is
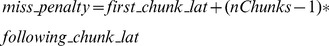
(2)and
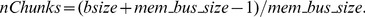
(3)


While the penalty to load each following memory block of the same basic block is
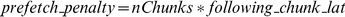
(4)and *nChunks* is also given by Equation 3. To distinguish the miss and prefetching latencies, a new cache line state named *IC_PREFETCH* is added to the cache model of Chronos to work together with the other two cache line state, *IC_HIT* and *IC_MISS*. For more information about the cache model in Chronos, please refer to [18].

### Effectiveness of BBIP-combined partial cache locking on WCET improvement

**Figure 7:**
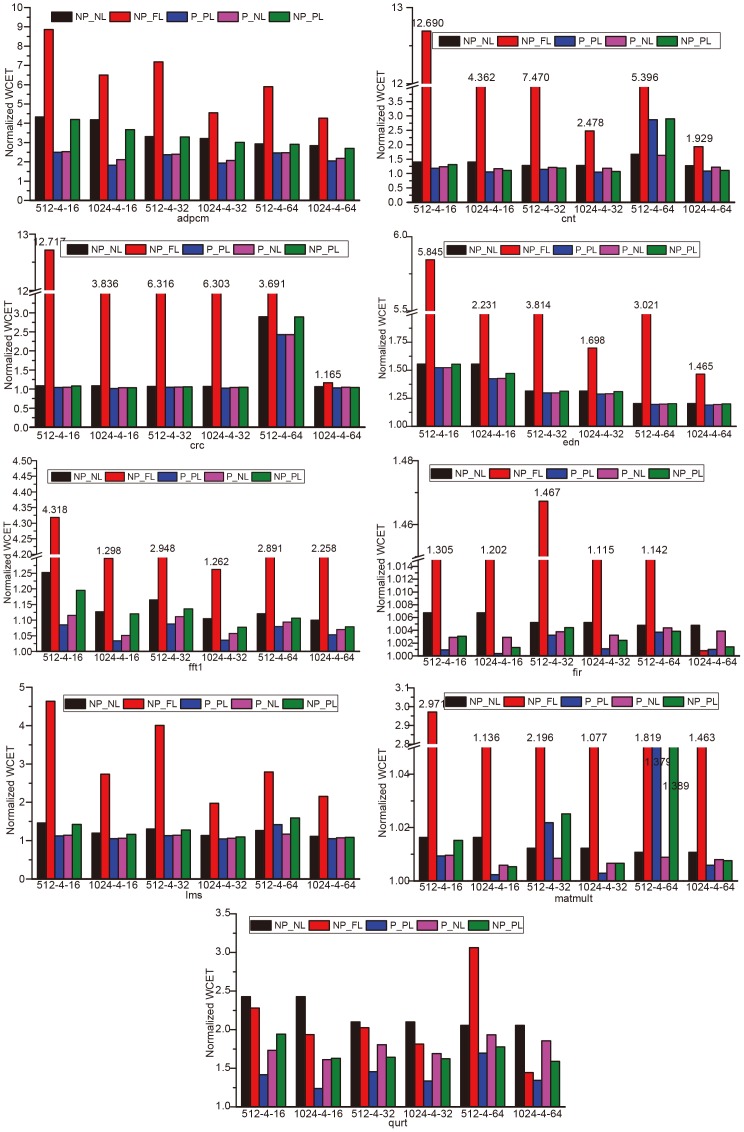
Normalized WCETs of the benchmarks in different configurations. The cache configuration is presented like *size-way-bsize*, where *size*, *way* and *bsize* represents the cache size, associativity of set and block size, respectively. So, the label 512-4-16 denotes a 512-byte 4-way associative cache with 16 blocks.

Figure 7 gives an overview of the WCET estimates for all the 9 benchmarks. For each benchmark, the WCETs are estimated in NP-NL, NP-FL, P-PL, P-NL and NP-PL for different cache sizes and different block sizes. As shown, for almost all benchmarks, P-PL has the lowest WCET value among all the cache configurations. Thus we say that P-PL provides good WCET improvement for all benchmarks in different cache configurations. In other word, our BBIP-combined partial cache locking methodology is effective in improving WCET. Moreover, we can see that, for each benchmark, the full cache locking represented as NP-FL provides high estimated values. That is to say, although full cache locking provides predictability to instruction cache, it increases the WCET of real-time applications, which poses negative effect on schedulability analysis of the system. This confirms our motivation to apply partial cache locking to improve WCET.

It is noted that, our results in Figure 7 may enlarge the disadvantage of full cache locking as we use a large cache miss penalty as shown in Table 5, compared to that used in [8]. In full cache locking, each access to an unlocked instruction will lead to an access to the memory, which results in a high slowdown and in turn increases the WCET. When more conservative memory access latency is used, such as 4 cycles, the WCET goes closer to that got in NP-NL. However, the relationship of WCETs between different configurations still holds.

According to the WCET estimates collected with static analysis (termed as NP-NL), the benchmarks can be classified into three categories:

- size sensitive: both cache size and cache block size affect the WCET estimate. The WCET estimate decreases as the cache and/or the cache block size become(s) larger. The benchmarks, *adpcm*, *fft1* and *lms*, fall into this category. Programs of this kind usually have a large program size (as shown in Table 6), which makes them gain from a bigger cache size. Meanwhile, a larger cache block exploits better spatial localities and thus more accesses are deemed as *always-hit*, which further improves the WCET estimate. The results also show that, the WCET estimates decrease more significantly for *fft1* and *lms* than *adpcm* as the cache size grows. As shown in Table 6, *adpcm* has the largest program size which is much larger (over 7 KB) than the cache size (512B or 1024B), while *fft1* and *lms* have a smaller size (about 2.4 KB and 2.7 KB respectively). The large program size makes *adpcm* not as sensitive as *fft1* and *lms* to the changing of the cache size. And more misses can be avoided for *fft1* and *lms* when the cache size grows to 1024-byte, which can be deduced from their WCET estimates. In NP-NL. Moreover, *fft1* and *lms* have a higher miss rate than *adpcm* when the cache size is 512-byte, as shown in Table 3, so more misses tend to be avoided due to the increase of the cache size.
- size partially sensitive: programs in this category (for example, *cnt* and *crc*) achieve some WCET improvements at some point where the cache size and cache block change. For *cnt* and *crc*, with 1024-byte cache being used, the WCET estimates decrease as the block size increases due to the fact more localities are exploited in a larger cache block. However, when a 512-byte cache is used, a sharp increase in the WCET estimates is observed when the cache block grows from 32-byte to 64-byte. This is due to the fact that there are some long distance jumps (or loops) which destroy the spatial localities in the instruction accesses. Loading a large cache block incurs more penalty (see Equation 2), while it will bring in very few cache hits as only a small part of the instructions are used before it is swapped out. We term the instructions in a block that are never accessed before it is swapped out as *zombie instructions*. When the cache block is small, this is not a big problem as the loading penalty is smaller and only few *zombie instructions* exist.
- size insensitive: programs in the category, including *edn*, *fir*, *matmult* and *qurt*, shows no improvement in the WCET estimates as the cache grows larger with the cache block being fixed. For *fir*, *matmult* and *qurt*, the program is small and most of the *hottest* basic blocks can be filled in a small (for example, 512 bytes) cache. For *edn*, the situation is different. According to Table 6, *edn* has a large program size, over 4K bytes. And there are many large basic blocks (see Table 1) which are *hot* in the worst-case execution and re-used again and again before being swapped out and never accessed again. So when the cache becomes larger, almost no extra conflict misses are avoided.

To show the advantage of BBIP-combined partial cache locking (P-PL) over static analysis (NP-NL) and full cache locking (NP-FL), we compare the normalized WCET values of them in the following discussions.

To evaluate the performance of partial cache locking, we estimate the WCET values of each benchmark with 1 to (*ns-1*) ways locked separately, where *ns* is the degree of associativity of the instruction cache. Then, the lowest one is picked as the WCET value of the program in NP-PL and P-PL, accordingly. We compare the WCETs of NP-PL and P-PL with those estimated with static analysis (NP-NL) and full cache locking (NP-FL) to show the WCET improvement.

To avoid misunderstanding, we state that, in the legends of the figures below, NP and P represent NP-PL and P-PL respectively. So, a legend like 1024-4-16:NP means a 4-way associative 1 KB cache with 16-byte blocks and no BBIP prefetching is used.

### WCET improvement of partial cache locking over static analysis

We discuss the WCET improvement of partial cache locking, both NP-PL and P-PL, over static analysis from the following two aspects:

1. We discuss the effect of different cache sizes on the WCET improvement with block size of the cache being fixed. In our discussion, we take cache configurations with 32-byte blocks as an example to explain.
2. We discuss the effect of different cache block sizes on the WCET improvement with the size of the cache being fixed. In our discussion, we use 1024-byte cache to carry out the discussions.

**Figure 8:**
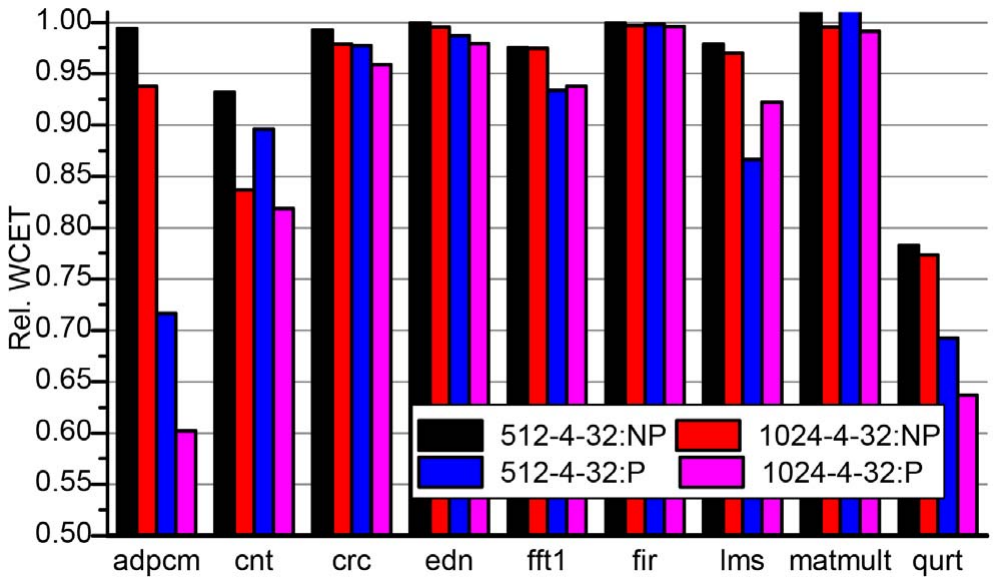
Relative WCETs after partially locking I-Cache without (a) and with (b) BBIP enabled to static analysis. The cache block size is fixed to 32-byte and the cache size is 512 and 1024 bytes,respectively. NP and P in the legends represent NP-PL and P-PL respectively.

**Figure 9:**
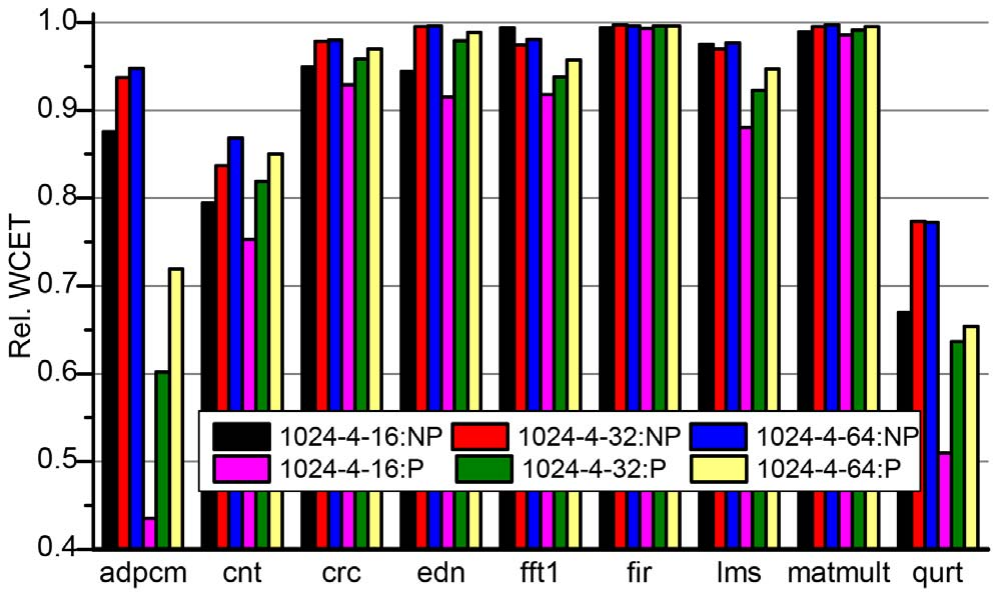
Relative WCETs after partially locking I-Cache without (a) and with (b) BBIP enabled to static analysis. The size of the cache is fixed to 1024-byte and the block size is 16, 32 and 64 byte, respectively.

Figure 8 and 9 show the relative WCETs of partial cache locking over static analysis for different cache configurations. They show the relative WCETs of the benchmarks after partially locking the instruction cache as the percentage of the WCETs without cache locking and BBIP. So, the 100% base line represents the WCETs of the benchmarks in NP-NL. A value below 100% means there is some WCET improvement over static analysis, otherwise, static analysis provides lower WCET estimates.

Let's first discuss the WCET improvement of NP-PL and P-PL over static analysis (NP-NL) with different cache size. In Figure 8, the left two bars for each program shows the improvement of only partial cache locking (NP-PL) over static analysis when the BBIP feature is disabled. While The other two bars for each program depict how much BBIP-combined partial cache locking improves the WCET over static analysis. Table 7 give the number of way(s) locked for each benchmark to achieve the highest improvement in NP-PL and P-PL, respectively.

**Table 7:** way(s) locked achieving the highest WCET improvement in NP-PL and P-PL.

NP-PL	512-4-16	1024-4-16	512-4-32	1024-4-32	512-4-64	1024-4-64
adpcm	1	3	1	3	1	3
cnt	2	3	2	3	2	3
crc	1	3	1	2	1	2
edn	1	3	1	2	1	2
fft1	2	3	1	1	1	2
fir	3	3	1	2	2	3
lms	1	1	1	1	1	1
matmult	1	3	1	3	2	2
qurt	3	3	3	2	2	3

P-PL	512-4-16	1024-4-16	512-4-32	1024-4-32	512-4-64	1024-4-64
adpcm	1	3	1	3	1	3
cnt	1	1	2	2	2	3
crc	1	3	1	2	1	2
edn	1	1	1	1	1	1
fft1	2	3	1	1	1	2
fir	1	2	1	1	2	2
lms	1	2	1	1	1	1
matmult	1	3	1	3	2	2
qurt	3	3	3	2	2	3

When BBIP is disabled, the partial locking mechanism (NP-PL) can improve the WCET over static analysis (NP-NL) for almost all benchmarks with different cache configurations. While the improvement varies among different programs. This can be explained as follows. On the one hand, in a partially locked cache, the contents locked in the cache avoid some mandatory misses (termed as *gain*). While, on the other hand, locking some ways means the ways available for block replacement are reduced, which may incur extra conflict misses (turned as negative effect). If the gain of cache locking is not significant enough to outweigh the negative effect, the improvement will be unimpressive, none or even negative. When the cache is small (512 bytes in our evaluation), the memory blocks are more likely to conflict with each other, which makes the WCET estimates more sensitive to the reduction of cache ways and the negative effect is non-ignorable. For *edn* and *fir*, the gain is almost neutralized by the negative effect, so the improvement is almost none in the 512-byte cache. For *adpcm*, which has a large program size, only a small percentage of its *hot* basic blocks can be locked and the gain is thus limited. And the negative effect is comparable to the gain of cache locking when the cache is small, so the improvement is unimpressive. For *fir*, which has the smallest program size, the WCET estimated with static analysis is perfect. So there is little space left for improvement and the improvement cannot be impressive even when the cache size is large. For *matmult*, which is also small and the WCET estimate is perfect, the negative effect outweighs the gain of cache locking and makes NP-NL a little better than NP-PL. The highest improvement is achieved for *qurt*. Two factors account for this. Firstly, although *qurt* is small, a high proportion of accesses are determined statically as *always-miss*, which makes the relative WCET estimate high (over 2) in NP-NL (see Figure 7) and a large space left to improve. Secondly, *qurt* is *size insensitive* according to our discussions for Figure 7, which makes the negative effect of cache locking unremarkable. With the block size fixed, such as 32-byte, the WCET improvement of NP-PL over NP-NL increases for almost all benchmarks as the cache size increases. When the cache grows larger, the negative effect of cache locking is reduced as fewer memory blocks conflict. Moreover, as more *hot* basic blocks are locked, more gains are achieved in the locked ways. These two factors accounts the higher improvement when the cache is 1024-byte for most programs. For *edn*, although it has a high relative WCET estimate in NP-NL, the improvement is not significant. This is due to the fact that, most of its hottest basic blocks in the worst-case execution are large and thus only a small part of them can be locked in the cache, which affects the WCET improvement. For programs which has perfect WCET estimates in NP-NL, such as *fir*, the improvement cannot be significant even in a large cache.

On average, about 5.63% and 9.54% improvement is achieved for programs without *perfect* WCET estimates in NP-NL when the cache is 512 and 1024 bytes, respectively.

Compared to NP-PL, P-PL provides better WCET improvement over static analysis for all benchmarks, which can be deduced from Figure 7. This is easy to understand. As the prefetching penalty (see Equation 4) is always smaller than the miss penalty (see Equation 2), a cache with BBIP enabled will provide at least as good performance as that without BBIP. As shown in Figure 8, the WCET improvement increases as the cache becomes larger for most programs, except *fft1* and *lms*. This is easy to explain. On the one hand, for most programs, the WCET estimated in NP-NL keeps almost unchanged or slightly reduced when the cache grows from 512-byte to 1024-byte and block size being 32-byte (see Figure 7). On the other hand, more basic blocks are able to be locked and a larger unlocked portion in a large cache can avoid more conflict misses. These two factors contribute to the more significant WCET improvement observed when a larger cache is used. For *fft1* and *lms*, the WCET in NP-NL decreases significantly as the cache size grows, while the WCET estimated in P-PL is not reduced so much, so the WCET improvement is not so significant. For *fir* and *matmult*, which leave little space for improvement as discussed above, the improvement is non-significant.

Compared to the Relative WCETs in NP-PL, the WCET of *adpcm* decreases sharply when BBIP instruction prefetching is enabled. This is because many basic blocks in *adpcm* benefit from the BBIP mechanism. While *edn* benefits little from the BBIP mechanism due to the fact that the large basic blocks are re-used before it is swapped out and almost never accessed again, which means the prefetching operation occurs rarely and only very limited extra cache misses for a basic block are saved during the execution.

On average, about 15.12% and 18.39% improvement is achieved for programs other than *crc*, *fir* and *matmult*, which have *perfect* WCET estimates in NP-NL, for 512-byte and 1024-byte caches, respectively.

Figure 9 shows the WCET improvement of NP-PL and P-PL over static analysis with different cache block size. All benchmarks in both Figure 9 show some WCET improvement, which decreases as the block size increases for most programs. This is due to the fact that a larger cache block exploits better spatial localities and more cache access are deemed as *always-hit* in the static analysis. Moreover, as the block size increases, fewer basic blocks are prefetched by the BBIP mechanism, which also increases the WCET values. The anomaly shown in NP-PL for *fftl* and *lms* is due to the fact that, when cache block size grows from 16-byte to 32-byte, locking some ways in the cache avoids more cache misses than that otherwise being left unlocked and analysed statically.

Table 8 shows the summary of the statistical analysis results of the relative WCETs of P-PL to NP-NL. The results are collected with OriginPro, a data analysis and graphing software [25]. In the calculation of the confidence intervals and the hypothesis testing, the significance level, denoted as 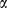, is set to 0.1. That is, in 90% cases, the relative WCETs will fall in the confidence intervals. The *test mean* values are the minimal values used in the hypothesis testing to make null hypothesis, which make the testing pass. For example, a *test mean* valued 0.8 means that, at the 0.1 level, the population mean is significantly less than 0.8, but not significantly less than a value smaller than 0.8, such as 0.79. As Table 8 shows, the *test mean* values are smaller than 1, except that in 512-4-64 (1.22). That is, in cache configurations other than 512-4-64, the P-PL provides some WCET improvement compared to the static analysis technology. The reasons why P-PL provides a larger WCET estimate than NP-NL in 512-4-64 are twofold. Firstly, as the cache size is small, only a few basic blocks can be locked and thus limited compulsory misses can be avoided, while the reduction of ways available in the unlocked ways results in significant conflicts. Secondly, a large cache block size benefits static analysis as more localities can be exploited, while it poses negative effect on BBIP as less memory blocks are prefetched.

**Table 8:** Statistic analysis results of the Rel. WCETs of P-PL to NP-NL.

Rel. WCET(P-PL/NP-NL)	512-4-16	1024-4-16	512-4-32	1024-4-32	512-4-64	1024-4-64
Sample Mean	0.84	0.813	0.898	0.871	1.074	0.897
Standard deviation	0.166	0.206	0.119	0.153	0.297	0.129
Confidence intervals ( 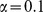 )	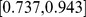	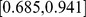	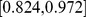	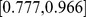	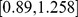	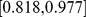
Test mean ( 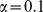 )	0.92	0.91	0.96	0.95	1.22	0.96

To sum up, our BBIP-combined partial cache locking mechanism indeed provides some WCET improvement over static analysis for different cache sizes and block sizes. Compared to the method using partial cache locking alone, it is better to combine BBIP with partial cache locking to improve the WCETs of the real-time applications.

### WCET improvement of partial cache locking over full cache locking

**Figure 10:**
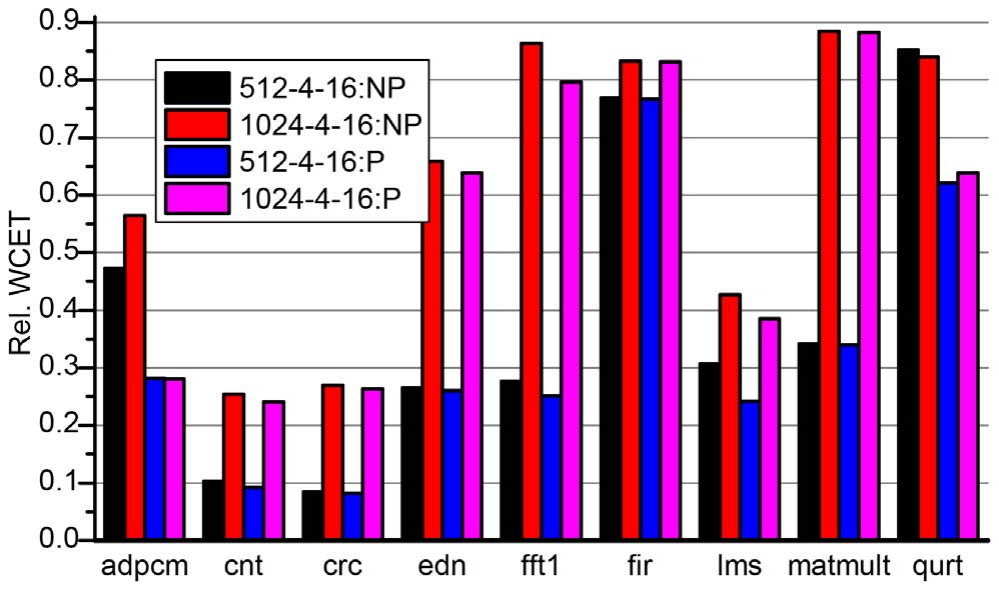
Relative WCETs after partially locking I-Cache without (a) and with (b) BBIP enabled to full cache locking. The cache block size is fixed to 16-byte and the cache size is 512 and 1024 byte, respectively. NP and P in the legends represent NP-PL and P-PL respectively.

**Figure 11:**
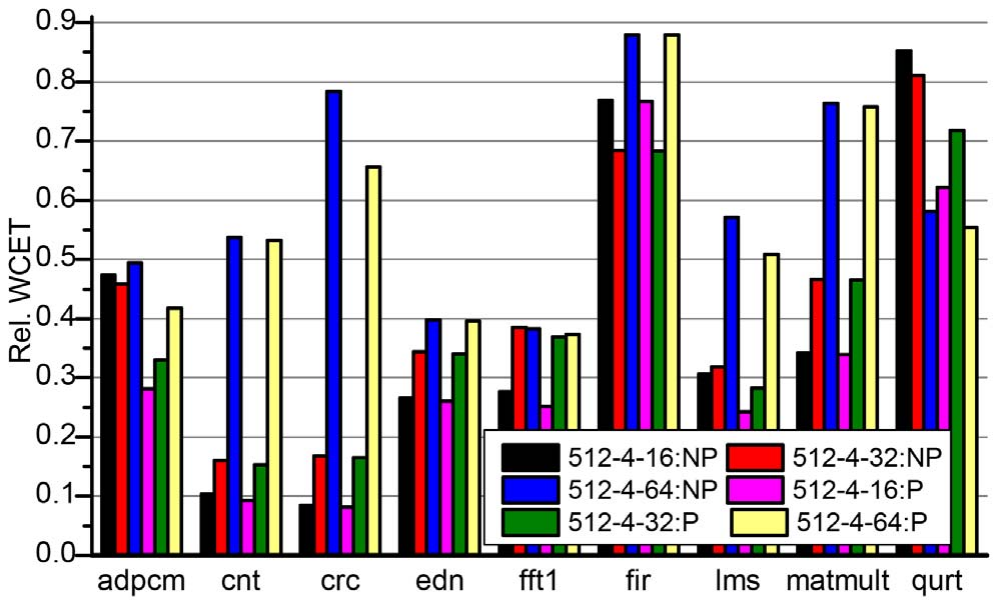
Relative WCETs after partially locking I-Cache without (a) and with (b) BBIP enabled to full cache locking. The size of the cache is fixed to 512-byte and the block size is 16, 32 and 64 byte, respectively.

Similar to the discussions above, we compare the WCETs of partial cache locking with and without BBIP with full cache locking [8]. The WCET improvement of both kinds of partial cache locking methods (NP-PL and P-PL) over full cache locking (NP-FL) is depicted in Figure 10 and 11.

The discussions also go in the following two ways:

1. The effect of different cache sizes on the WCET improvement is discussed with the block size of the cache being fixed. As we have used cache configurations with 32-byte blocks above, here cache configurations with 16-byte blocks are used as examples to discuss. The results are shown in Figure 10.
2. The effect of different cache block sizes on the WCET improvement is discussed with the size of the cache being fixed. Instead of 1024-byte caches, we use 512-byte cache to carry out the discussions. The results are shown in Figure 11.

For all benchmarks, the partial cache locking (both NP-PL and P-PL) mechanisms show significant WCET improvement over the full cache locking (NP-FL) mechanism in both 512-byte and 1024-byte caches. And the improvement decreases as the cache size grows from 512-byte to 1024-byte for all programs except *qurt* in NP-PL. When the cache becomes larger, more valuable basic blocks are locked and the WCETs decrease dramatically as shown in Figure 7. Meanwhile, the WCETs of NP-PL and P-PL have already dropped to a low level when the cache is 512-byte, and the extra WCET reductions that result from the further increase of cache size are not so significant. For *qurt*, the improvement even improves a little when the cache size increases. In *qurt*, the reduction of WCET estimates in NP-FL is not as significant as that in NP-PL (see Figure 7) due to the high miss penalty in the fully locked cache.

Comparing the programs' relative WCETs of the same cache configuration in NP-PL and P-PL in Figure 10, it is clear that *crc*, *fir* and *matmult* have almost the same WCETs in NP-PL and P-PL. This is because the three programs have small program size, and the cache access conflicts in the unlocked portion of the cache are rare and thus very few cache misses are avoided by BBIP. From Figure 7, we can see these programs have *perfect* WCET estimates in NP-NL, which means they are little space left for improvement. On average, NP-PL reduces 61.41% and 37.83% of the WCET estimates of NP-FL for 512-byte and 1024-byte caches, respectively. And the corresponding WCET improvement of P-PL over NP-FL is 67.22% and 44.90%, respectively.

As shown in Figure 11, the effect of cache block size on WCET improvement varies for different programs. The improvement decreases with the increase of the cache block size for most programs, except *adpcm*, *fir* and *qurt* in NP-PL. In our evaluation, a cache-line-size buffer is used in NP-FL to save some accesses to the main memory. As the buffer is always of the same size as a cache line, extra high-penalty accesses to the main memory are saved when the cache block size increases. Although NP-PL and P-PL also benefit from the increase of the cache block due to the increased spatial localities, it is not as sensitive as that in NP-FL as the WCET estimates in NP-PL are usually much smaller than that in NP-FL and less space is left for improvement. The improvement of *adpcm* shows very little difference in NP-PL when the cache block size changes due to the fact that it has a very large program size compared to the size of the cache and the changing of spatial localities in cache blocks with different sizes does not affect the cache performance too much. The WCET estimate of *fir* is very close to that in a perfect I-Cache (which we call it *perfect*) in cache configurations other than NP-FL. So the relative WCET values only present the changing of the WCET estimates collected in NP-FL. From Figure 7, we can see that there is a sudden increase of the WCET estimate for *fir* in NP-FL when the cache block size is 32-byte. This is because *fir* has a very small program size, only 17 basic blocks, the content selection algorithm has every limited candidates to be selected to lock to achieve the optimal performance. This explanation also holds for the behavior in P-PL for *fir*. A very high WCET estimate is collected for *qurt* in NP-FL when a 512-byte cache with 64-byte blocks are used. This is because many conflicts exist between the most *valuable* basic blocks in the worst-case execution and locking a large block causes some less *valuable* contents also being locked. The anomaly disappears in a 1024-byte cache where almost all the most *valuable* blocks are able to be locked. Compared to NP-PL, P-PL shows a similar trend for most programs. While, a dramatic drop of the WCET estimates is observed for *adpcm* and *qurt* when a 16-byte block is used. This is because both of them benefit a lot from BBIP when the cache block is small.

As shown in Figure 7, *fir* shows *perfect* WCET estimates in cache configurations other than NP-FL. And it shows high WCET estimates in NP-FL for all cache configurations except 1024-4-64. Considering the fact that there are only 74 static instructions in the program, it is quite counter-intuitive. This can be explained as follows. Although the program is small, it cannot be locked totally in the cache, especially taking the block mapping in the cache into consideration. Each access to an unlocked instruction will either hit in the *buffer* or lead to an access to the main memory. When the cache is 512-byte, more instructions are left unlocked either because the size is not large enough or there is a mapping conflict. And the size of the *buffer* affects the number of access to the main memory, which has a high penalty. A large *buffer* usually avoids some memory accesses if there are some spatial localities existing in the access. The number of unlocked instructions and the penalty to access each unlocked instruction together determine the worst-case cache performance of the program. When the unlocked instructions are rare and most of them hit in the *buffer*, which is true in 1024-4-64, the WCET estimate for NP-FL is small, about 1.003 compared to that in the perfect cache.

Table 9 shows the summary of the statistical analysis results of the relative WCETs of P-PL to NP-FL. The results are also collected with OriginPro. The significance level, denoted as 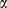, is also set to 0.1. The results show that, our BBIP-combined partial cache locking mechanism provides significant WCET improvement over static analysis for different cache sizes and block sizes.

**Table 9:** Statistic analysis results of the Rel. WCETs of P-PL to NP-FL.

Rel. WCET(P-PL/NP-FL)	512-4-16	1024-4-16	512-4-32	1024-4-32	512-4-64	1024-4-64
Sample Mean	0.327	0.551	0.39	0.632	0.564	0.701
Standard deviation	0.228	0.261	0.201	0.26	0.172	0.211
Confidence intervals ( 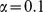 )	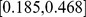	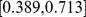	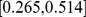	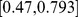	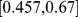	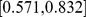
Test mean ( 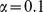 )	0.44	0.68	0.49	0.76	0.65	0.8

As a result, we conclude that our BBIP-combined partial cache locking mechanism is effective in improving the WCET estimates, compared to the full cache locking mechanisms. Also, compared to the method using partial cache locking alone, it is better to combine BBIP with partial cache locking to improve the WCETs of the real-time applications.

### Contributions of BBIP and partial cache locking to WCET improvement

From the discussions above, we can conclude that partial cache locking, especially that combined with BBIP, indeed improves the WCET of the programs compared to static analysis and full cache locking. In this section, we will show the contributions of BBIP and partial cache locking to the WCET improvement provided by P-PL.

Table 10 shows the relative WCETs of P-NL and NP-PL to P-PL. The 100% base line represents the WCETs obtained in P-PL. A value below 100% means the WCET is smaller than that obtained with both BBIP and partial cache locking being used. Otherwise, the WCET is bigger.

**Table 10:** The relative WCETs of P-NL and NP-PL to P-PL. The base line 100% is the WCET obtained in P-PL.

P-NL/P-PL	512-4-16	1024-4-16	512-4-32	1024-4-32	512-4-64	1024-4-64
adpcm	1.014	1.157	1.008	1.075	1.004	1.064
cnt	1.048	1.108	1.061	1.128	***0.569***	1.128
crc	1.006	1.024	1.006	1.015	1.002	1.017
edn	1.001	1.001	1.001	1.002	1.001	1.003
fft1	1.028	1.016	1.021	1.020	1.014	1.016
fir	1.002	1.003	1.001	1.002	1.000	1.003
lms	1.015	1.009	1.013	1.015	***0.824***	1.018
matmult	1.001	1.004	0.986	1.004	***0.732***	1.002
qurt	1.222	1.304	1.241	1.266	1.142	1.379
Average	1.037	1.070	1.038	1.058	0.921	1.070
standard deviation	0.071	0.104	0.079	0.089	0.178	0.123
Confidence intervals ( 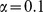 )	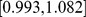	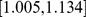	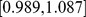	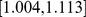	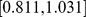	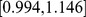

As shown in the upper half of Table 10, most of the values are above 1, which means P-PL provides some WCET improvement over P-NL. In other words, whether significant or not, partial cache locking contributes to the WCET improvement. Moreover, for a given cache block size, the relative WCET usually increases as the cache becomes larger. This is because, a bigger cache can avoid some conflict misses that otherwise exists in a smaller cache due to the decrease of the available way in the static analysis. In our estimation, most programs shows non-significant WCET improvement, except *adpcm* and *qurt*. This is because, most programs used here are small, and most of the instruction accesses in them can be analyzed statically. For *adpcm*, which has the largest program size (see Table 6), most of the *hottest* basic blocks in the worst-case execution are small and thus more basic blocks can be locked and fewer are prefetched. This explains why it has relatively high improvement. The maximum improvement for *adpcm* is about 15.7%. The reason why the small program, *qurt* as shown in Table 6, has high improvement is quite different. Firstly, *qurt* has a smaller program size, so it is less sensitive to the decrease of the available ways in the static analysis than *adpcm*. This can be deduced from Table 7, where more ways are locked to get the best partial lock performance for *qurt* than *adpcm* when the cache is small. Secondly, most of the *hottest* basic blocks in *qurt* are very small, covering only 1 or 2 memory blocks when a 16-byte cache block is used. This limits the instruction prefetching and benefits the cache locking. The maximum improvement is about 37.9% for *qurt*.

Compared to P-NL, P-PL shows high WCET estimates in some cache configurations, such as those underlined and marked bold in Table 10. When a 512-byte cache with 64-byte blocks is used, *cnt*, *lms* and *matmult* show much lower WCET estimates when only BBIP is enabled. These three programs are very sensitive to the decrease of the available ways when the cache is small. In our evaluation, a lot of extra conflict misses are observed when the degree of association is reduced by 1. For example, the WCET estimated with static analysis for a 3-way 384-byte cache with 64-byte blocks is about 3 times larger than that in NP-PL for a 4-way 512-byte cache with 64-byte blocks. This can also be deduced from the high WCET values in NP-PL collected with the cache configuration being 512-4-64 in Figure 7. When partial cache locking is performed on these programs, the available ways in the unlocked portion decrease (smaller than 4) and the WCET becomes much larger.

We have to emphasize that, although only a little WCET improvement is observed for most programs in Table 10 when BBIP is combined with partial cache locking, we believe it is a good idea to do so. The cache locking mechanism brings in predictability and BBIP improves WCET. Combining these two can make a good balance between predictability and lower WCET estimate.

From the bottom half of Table 10, we can conclude that in any case, P-PL will provide at least as good WCET improvement as NP-PL. The improvement is more obvious when the program is larger. Moreover, P-PL works well for most programs when a small cache with small blocks is adopted. This is because BBIP is more efficient when the cache block size is small as a lot of basic blocks gain from BBIP. For *adpcm*, the relative WCET is almost 2 when the cache configuration is 1024-4-16.

The hypothesis testing results show that, at the 0.1 level (eq. The significance level 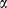 is 0.1), the mean of the relative WCETs of NP-PL and P-NL to P-PL is *significantly* greater than 1.08 and 1.03, respectively. Then we can say, both partial cache locking and BBIP contribute to the WCET improvement in P-PL.

To sum up, BBIP-combined partial cache locking will take advantages of both BBIP and partial cache locking to improve WCET. It will provide better performance when a more complicated program in real-life is used. Moreover, our partial cache locking mechanism is especially effective in a system with an instruction cache with small blocks.

### Gain of implementing BBIP in hardware

According to Table 1, 64 items in BBIT are enough to record the basic block information for most benchmarks. For *adpcm*, which has a large number of basic blocks, we can pick the most *valuable* 64 basic blocks to record in BBIT. How to select the *valuable* basic blocks to record in BBIT is out of the scope of the paper, we do not discuss it here. We assume each item in BBIT is 4-byte long, then the total cost of BBIT is 256-byte. So a 512-byte cache with BBIP equipped costs about 768-byte in hardware. And if it provides lower WCET estimates than that collected with static analysis in a cache whose size is 768-byte or larger, we can say it is worth implementing BBIP in hardware. We compare the WCET values collected in 512-byte BBIP-enabled (P-NL) caches with that calculated by static analysis on 1024-byte caches (see Figure 7) to show the effectiveness of BBIP in reducing the WCET estimate. The results show that, when the block size is 32 bytes, 6.42% WCET improvement is achieved on average. For large programs like *adpcm*, BBIP is very effective in reducing the WCET estimate and the improvement is about 34.33%.

### Some discussions

In our experiments, we find that combining the BBIP mechanism with partial cache locking indeed improves WCET. In almost all cases, our partial cache locking mechanism outperforms the static analysis and full cache locking method. Considering the content selection algorithm, we find that our greedy algorithm selects the most *profitable* basic blocks for most programs. But for some benchmarks, such as *cnt*, which have a small number of *hot* basic blocks, the algorithm may waste some cache blocks for a large cache due to cache mapping conflict or locking *useless* basic blocks. We believe that when the benchmark is more complicated or the cache content selecting is performed in a multi-tasking scene, the performance of the algorithm will be more impressive.

## Conclusions and Future Work

In this paper, we try to combine the BBIP mechanism [19] and partial cache locking to improve WCETs of real-time applications. We have proposed a two-phase content selection algorithm to select the *hottest* basic blocks greedily. To evaluate the effectiveness of our BBIP-combined partial cache locking mechanism in reducing the WCETs of real-time programs, we compare its WCETs with that collected in a system where the cache is statically analysed and fully locked, respectively. The results show that it indeed provides some WCET improvement over static analysis and full cache locking. Moreover, we also discuss the contributions of BBIP and partial cache locking on our methodology. The results show that applying BBIP and partial cache locking together provides lower WCETs than applying BBIP or partial cache locking alone in most cases.

Although we have done plenty of work in combining the BBIP mechanism with partial cache locking, there are also some imperfections in our work which will be addressed in our future work. Firstly, all basic blocks are selected statically without considering the change of the worst-case execution path. Although our content selection algorithm provides a safe WCET estimation with the support of the Chronos framework, the WCET improvement may not be optimal when the worst-case execution path changes. Secondly, we do not provide prediction mechanisms to statically determine the number of ways locked in order to get the best performance. All the aspects will be considered in the future. Moreover, as future work, we will extend our partial locking mechanism to multi-tasking real-time systems.
